# Dyslipidemia and Obesity in Ischemic Stroke

**DOI:** 10.7759/cureus.45409

**Published:** 2023-09-17

**Authors:** Bikram P Gajurel, Anju Gurung, Rajeev Ojha, Reema Rajbhandari, Ragesh Karn

**Affiliations:** 1 Neurology, Tribhuvan University Institute of Medicine, Kathmandu, NPL; 2 Internal Medicine, Tribhuvan University Institute of Medicine, Kathmandu, NPL; 3 Neurology, Tribhuvan University Teaching Hospital, Kathmandu, NPL

**Keywords:** nepal, gender, diabetes mellitus, central obesity, dyslipidemia, ischemic stroke

## Abstract

Background

Dyslipidemia and obesity are both important risk factors for the first and recurrent ischemic strokes. Dyslipidemia is highly prevalent among Asian populations, and the prevalence of obesity is also noted to be progressively increasing in this population. This study was carried out to determine the prevalence of dyslipidemia and central obesity and their association with each other and various cardiovascular risk factors among patients who presented with an acute ischemic stroke to a tertiary care university hospital in Nepal.

Methods

This study is a secondary analysis done on data from a prospective observational study that was carried out on patients who were either acutely admitted to or visited the outpatient department of the hospital with a diagnosis of ischemic stroke. Dyslipidemia was defined according to the third report of the National Cholesterol Education Program expert panel on detection, evaluation, and treatment of high blood cholesterol in adults. Obesity was defined as central obesity by measuring waist circumference. Data were collected by convenience sampling and analyzed by IBM Corp. Released 2019. IBM SPSS Statistics for Windows, Version 26.0. Armonk, NY: IBM Corp. Significant variables were compared with logistic regression analysis. Other data were expressed as frequencies and percentages.

Results

Out of 145 patients, 77 were male (53.1%). The mean age of the patients was 60.15 years. Dyslipidemia and central obesity were present in 96.6% and 57.9% of the patients, respectively. The most common lipid abnormality was low-density lipoprotein cholesterol, present in 82.8% of the patients, followed by high triglycerides, present in 21.4% of them. Dyslipidemia was not associated with any vascular risk factors. Central obesity was significantly associated with female gender, diabetes, and low-density lipoprotein cholesterol in univariate analysis. However, in multivariate logistic regression analysis, it was significantly associated with only female gender (p=0.003) and diabetes (p=0.012).

Conclusion

Dyslipidemia and central obesity are very common in patients with ischemic stroke. Dyslipidemia is not associated with any vascular risk factors. However, central obesity is significantly associated with the female gender and diabetes.

## Introduction

Dyslipidemia is a major risk factor for ischemic stroke and its recurrence [1]. High levels of total cholesterol (TC) and low-density lipoprotein (LDL) both predispose to atherosclerotic ischemic stroke [1]. Studies have also found an association between low levels of high-density lipoprotein (HDL) and elevated triglyceride (TG) levels and ischemic stroke [2,3]. Obesity is another important risk factor implicated in the causation of stroke in both genders, independent of other major cardiovascular risk factors [4-6].

Studies have shown that Asian populations have a higher prevalence of lipid abnormalities as compared to non-Asian populations [7]. They tend to have a higher prevalence of low HDL cholesterol and elevated triglycerides and a lower prevalence of high serum cholesterol than non-Asian populations [8]. This particular pattern of dyslipidemia is also called atherogenic dyslipidemia because of its association with atherosclerotic cardiovascular diseases [8]. With changing lifestyles and living standards, the prevalence of overweight and obesity has been rising rapidly in the Indian subcontinent compared to other parts of the world [9].

We carried out this research to determine the prevalence and types of dyslipidemia as well as the prevalence of central obesity in patients who presented with acute ischemic stroke at a tertiary care university hospital in Kathmandu, Nepal. This study also examined the association of dyslipidemia, central obesity, and various vascular risk factors in the same patient population.

## Materials and methods

This research is a secondary analysis done on data from a prospective observational study that was carried out to study the association of vascular risk factors and carotid atherosclerosis in patients who were at least 18 years of age and were either acutely admitted to the hospital or visited the outpatient neurology department of Tribhuvan University Teaching Hospital, Kathmandu, Nepal, with a diagnosis of acute ischemic stroke. The diagnosis of ischemic stroke was made clinically and was supported by radiological studies that included computed tomography or magnetic resonance imaging of the brain. Dyslipidemia was defined based on fasting serum lipid profile values according to the third report of the National Cholesterol Education Program (NCEP) Expert Panel on Detection, Evaluation, and Treatment of High Blood Cholesterol in Adults (ATP III): any one of total cholesterol (TC) ≥ 200 mg/dL; low-density lipoproteins (LDL) ≥ 130 mg/dL; triglycerides (TG) ≥ 150 mg/dl; and high-density lipoproteins (HDL) < 40 mg/dL in men and < 50 mg/dL in women [10]. Serum lipid levels were measured within one or two days of admission. As this study is a secondary analysis, disorders that could secondarily lead to a rise in lipid levels could not be excluded. To diagnose central obesity, the waist circumference was measured at the midpoint between the lower margin of the last palpable rib and the top of the iliac crest at the end of a normal expiration, with the arms relaxed at the sides [11]. In patients in whom the measurements could not be taken in a standing position, they were taken in the supine position. In a study done to find the difference between supine and standing abdominal circumference in adults, supine abdominal girth measurement (done manually or estimated on computed tomography images) and standing measurements done manually were equivalent to abdominal circumferences up to 110 cm [12]. Central obesity was defined as a waist circumference (WC) of ≥ 90 cm in males and ≥ 80 cm in females, according to the clinical practice guidelines for the evaluation and management of obesity in India published by the Endocrine Society of India [13]. The waist-to-hip ratio was not used to measure obesity in this study, as we could not find any study that could address the equivalence of standing and supine measurements of hip circumferences.

Data collection was done only after patients or their caregivers provided informed consent and took place from December 2020 to November 2021. The required minimum sample size for the primary research was calculated as 145 based on the population prevalence of ischemic stroke of 36.5% among neurological admissions in hospital settings at a 5% margin of error and subsequently corrected for a finite population size of a single center [14]. Convenience sampling was used to select the patients. The collected data were entered into and analyzed using the IBM Corp. Released 2019. IBM SPSS Statistics for Windows, Version 26.0. Armonk, NY: IBM Corp.

The associations were analyzed using the Chi-square test and Fisher’s exact test. Significant variables were compared with logistic regression analysis. Other data were expressed as frequencies and percentages. The proposal to carry out the primary research and study the variables was approved by the Institutional Review Committee of the Institute of Medicine, Tribhuvan University, Kathmandu, Nepal [IRC number: 185(6-11) E2;077/078].

## Results

Out of 145 patients, 77 were male (53.1%). The mean age of the patients was 60.15 years (± 16.5 years, range 19-92 years). Dyslipidemia was present in 140 (96.6%) of the patients. Central obesity was present in 84 (57.9%) of the patients. Total cholesterol ≥ 200 mg/dL was present in 11 (7.6%), LDL ≥ 130 mg/dL was present in 13 (9%), triglycerides ≥ 150 mg/dl were present in 31 (21.4%), and low HDL was present in 120 (82.8%) of the patients (HDL < 40 mg/dL in 56 out of 77 men and HDL < 50 mg/dL in 64 out of 68 women). The summary of different lipid types is presented in Table 1.

**Table 1:** Summary of different lipid types.

Lipid Types	Minimum (mg/dL)	Maximum (mg/dL)	Mean (mg/dL)	Standard Deviation
Total Cholesterol	70	251	145.97	33.64
Low-Density Lipoprotein	31	182	83.57	30.38
High-Density Lipoprotein	19	81	38.1	8.33
Triglyceride	43	354	117.21	61.67

The association of dyslipidemia with different variables, the association of central obesity with different variables, and the logistic regression analysis of variables associated with central obesity are shown in Tables 2-4.

**Table 2:** Association of dyslipidemia with different variables. *Calculated by using the Fisher’s exact test.

Variables		Dyslipidemia		p-value*
		Yes (n=140) (%)	No (n=5) (%)	
Gender	Female	68 (48.6%)	0 (0%)	0.061
	Male	72 (51.4%)	5 (100%)	
Age > 50 years	Yes	99 (70.7%)	4 (80%)	1.00
	No	41 (29.3%)	1 (20%)	
Hypertension	Yes	49 (35%)	4 (80%)	0.06
	No	91 (65%)	1 (20%)	
Diabetes	Yes	26 (18.6%)	1 (20%)	1.00
	No	114 (81.4%)	4 (80%)	
Smoking	Yes	83 (59.3%)	3 (60%)	1.00
	No	57 (40.7%)	2 (40%)	

**Table 3:** Association of central obesity with different variables. *Calculated by using Chi-square and Fisher’s exact (when numbers in a cell are less than 5) tests.

Variables		Central Obesity		p-value*
		Yes (n=84) (%)	No (n=61) (%)	
Gender	Female	49 (58.3%)	19 (31.1%)	0.001
	Male	35 (41.7%)	42 (68.8%)	
Age > 50 years	Yes	57 (67.9%)	46 (75.4%)	0.322
	No	27 (32.1%)	15 (24.6%)	
Hypertension	Yes	30 (35.7%)	23 (37.7%)	0.806
	No	54 (64.3%)	38 (62.3%)	
Diabetes	Yes	21 (25%)	6 (9.8%)	0.021
	No	63 (75%)	55 (90.2%)	
Smoking	Yes	48 (57.1%)	38 (62.3%)	0.533
	No	36 (42.9%)	23 (37.7%)	
Dyslipidemia	Yes	82 (97.6%)	58 (95.1%)	0.650
	No	2 (2.3%)	3 (4.9%)	
High Total Cholesterol	Yes	7 (8.3%)	4 (6.6%)	0.761
	No	77 (91.7%)	57 (93.4%)	
High Low-density Lipoprotein	Yes	10 (11.9%)	3 (4.9%)	0.238
	No	74 (88.1%)	58 (95.1%)	
High Triglycerides	Yes	20 (23.8%)	11 (18%)	0.402
	No	64 (76.2%)	50 (82%)	
Low High-density Lipoproteins	Yes	74 (88.1%)	46 (75.4%)	0.0458
	No	10 (11.9%)	15 (24.6%)	

**Table 4:** Logistic regression analysis of variables associated with central obesity.

Variables	Odds Ratio	95% Confidence Interval	p-value
Low High-Density Lipoproteins	1.782	0.689 - 4.611	0.234
Female Gender	3.091	1.474 - 6.481	0.003
Diabetes	3.759	1.344 - 10.517	0.012

## Discussion

The prevalence of dyslipidemia in 96.6% of the study population is notably higher compared to similar prior studies in Nepal. In a recent study published by another center in Nepal, the prevalence of dyslipidemia based on the same criteria as this study was only 46.05% among patients with ischemic strokes [15]. Of note, that study had only 76 participants and did not include patients with cardioembolic strokes. Another study conducted among 150 diagnosed cases of ischemic strokes (including cardioembolic strokes) showed a prevalence of dyslipidemia of 80% based on the same criteria [16]. 

In a descriptive cross-sectional study conducted among patients presenting with acute coronary syndrome in Nepal, dyslipidemia-defined in a similar manner-was present in 48.6% of the participants [17]. In this study, high cholesterol and triglyceride were seen in 34 (32.4%) each, high LDL in 22 (21%), and low HDL in 31 (29.5%). In another large study done among patients presenting with ischemic stroke at a tertiary care center in Nepal, high total cholesterol was found in 64 (53.33%) participants, high triglycerides in 70 (58.33%), high LDL in 54 (45.00%), and low HDL in 51 (42.50%) [16]. These results appear quite different from ours, where elevated TC, TG, and LDL were present in a lower proportion of patients, but low HDL was present in a higher proportion (82.8%). Nepal has rich geographic and ethnic diversity. People living in different regions can have significant differences in physical activity and dietary habits, as well as genetic predispositions. These factors may have contributed to different lipid profile results in different patient samples from different parts of the country.

Typical abnormal lipid profiles in stroke and cardiovascular pathology show elevated levels of total cholesterol, LDL and triglyceride levels, and low levels of HDL cholesterol [18]. Multiple studies have shown that high levels of total cholesterol and LDL increase the risk of ischemic stroke [19], as do low levels of HDL [2]. Elevated levels of triglycerides also increase the risk of ischemic stroke [3]. In our study, the proportion of patients with high total cholesterol and LDL was not very high (7.6% and 9%, respectively). Compared to this, the proportion of patients with elevated TG was slightly higher (21.4%). However, the proportion of patients with low HDL was strikingly high (82.8%).

Even though dyslipidemia is an independent risk factor for ischemic cerebrovascular diseases, we tried to see if there was any association between dyslipidemia and other vascular risk factors in this study. We did not find a significant association between dyslipidemia and conventional vascular risk factors in our study (Table 2).

Analysis of the data from the original Framingham cohort has indicated that obesity is a significant and independent predictor of cardiovascular diseases [6]. In large prospective cohort studies, increased body mass indices have been found to be significantly associated with a greater incidence of stroke in both men and women [6,7]. In one published piece of literature from Nepal, the prevalence of overweight and obesity in the general population using the Asian-specific body mass index (BMI) cutoffs was 26.4% and 11.0%, respectively [20]. When the World Health Organization recommended BMI cutoffs were used, 18.2% of the people were overweight, and 4.3% were obese [20]. The prevalence of obesity in 57.9% of the participants in our study is expected to be higher than that in the general population. Of note, though, we used central obesity values, which have been shown to have better clinical utility than BMI in identifying individuals with cardiac and metabolic risk factors [21]. Studies have also shown that BMI measurements may not be a good predictor of risk of death and cardiovascular diseases compared to measurements of abdominal obesity [22]. 

Multiple prior studies have shown that obesity is often associated with hypertension and cardiovascular diseases, leading to adverse cardiovascular health outcomes [23]. Obesity is also known to increase the risk of type 2 diabetes mellitus [24]. The most prominent lipid disorder in obesity has been found to be low HDL. Multiple epidemiological studies have shown that there is a strong inverse relationship between HDL levels and obesity [25,26]. The prevalence of obesity has also been found to be higher in women than in men [27]. Our study showed that there was a statistically significant association of female gender, diabetes, and low HDL with central obesity based on univariate analysis (Table 3). However, multivariate analysis of our data showed that central obesity is associated with only female gender and diabetes (Table 4), compared to univariate analysis (Table 3), where it was also implicated in low HDL levels.

Obesity is more often present in women because of various hormonal and social factors [27]. An important biological aspect that plays a crucial role in female obesity is the occurrence of certain hormones during pregnancy and breastfeeding that prompt higher food consumption to ensure sufficient energy and fat storage, which is a natural physiological adjustment aimed at maintaining energy balance [27]. It may also result from their restrictive dietary patterns and lifestyles, as women often face challenges in accessing nutritious food and have limited opportunities to engage in physical activity and exercise outside of their daily chores [27,28].

Both diabetes and obesity can be considered epidemics due to their high and steadily increasing prevalence. Obesity not only contributes to the development of type 2 diabetes but also plays a major role in the progression of its complications [29]. Moreover, there is growing scientific evidence linking obesity and overweight to type 1 diabetes as well [29]. Weight gain can be an important complication of insulin treatment in both types of diabetes and can have a significant impact on increasing cardiovascular risks [29]. Although there is some evidence suggesting the involvement of gut microflora in the development of obesity and diabetes, this concept has not yet gained widespread acceptance [29].

Our study is a small study based on a single center done over a period of one year. Thus, the findings of this study may not be an exact representation of the actual prevalence of dyslipidemia and obesity in patients with ischemic stroke in the Nepalese population in general. The most important limitation of our study is the fact that we could not exclude other disorders like hypothyroidism or renal failure that could lead to dyslipidemia. This could be the reason behind the very high prevalence of dyslipidemia. We also could not exclude patients who were already on statins for a longer period of time. This study also does not mention whether dyslipidemia was already present or newly diagnosed, and other etiologies of ischemic stroke have not been mentioned. Another important limitation could be the reference that we used to justify the equivalence of supine and erect waist circumferences in our population. We used the data from the Spanish population [12], as we could not find studies addressing supine and erect waist circumferences in our population. The reference study did not discuss whether a waist circumference of more than 110 cm could be considered equivalent in supine and erect positions. We felt this was not significant for our study as this measurement is already suggestive of obesity, and our study is only concerned with the presence or absence of obesity rather than the actual quantitative measurement. A large-scale prospective study addressing all these limitations can be done across multiple centers in Nepal, and this study can serve as a pathway to carry out such studies in the future. 

## Conclusions

Dyslipidemia is very common in patients with ischemic stroke; the most common lipid abnormality is low levels of high-density lipoprotein cholesterol, followed by elevated levels of triglycerides. Central obesity is also very common in patients with ischemic stroke. This study showed that central obesity is significantly associated with the female gender and diabetes. In addition, based on our univariate analysis, it was also associated with low levels of high-density lipoprotein cholesterol. Future studies encompassing larger patient populations and lasting longer durations will be helpful to further elucidate the role of these risk factors in ischemic cardiovascular morbidities.
